# Isolation and Propagation of Human Corneal Stromal Keratocytes for Tissue Engineering and Cell Therapy

**DOI:** 10.3390/cells11010178

**Published:** 2022-01-05

**Authors:** Nur Zahirah binte M. Yusoff, Andri K. Riau, Gary H. F. Yam, Nuur Shahinda Humaira binte Halim, Jodhbir S. Mehta

**Affiliations:** 1 Tissue Engineering and Cell Therapy Group, Singapore Eye Research Institute, Singapore 169856, Singapore; zahirahmy@gmail.com (N.Z.b.M.Y.); andri.kartasasmita.riau@seri.com.sg (A.K.R.); nuur.shahinda.h.halim@seri.com.sg (N.S.H.b.H.); 2 Ophthalmology and Visual Sciences Academic Clinical Programme, Duke-NUS Medical School, Singapore 169857, Singapore; 3 Department of Ophthalmology, University of Pittsburgh, Pittsburgh, PA 15213, USA; gary.yam@pitt.edu; 4 Corneal and External Eye Disease Department, Singapore National Eye Centre, Singapore 168751, Singapore; 5 School of Materials Science and Engineering, Nanyang Technological University, Singapore 639798, Singapore

**Keywords:** keratocytes, corneal stroma, morphology, cell therapy, fibroblasts, serum

## Abstract

The human corneal stroma contains corneal stromal keratocytes (CSKs) that synthesize and deposit collagens and keratan sulfate proteoglycans into the stromal matrix to maintain the corneal structural integrity and transparency. In adult corneas, CSKs are quiescent and arrested in the G0 phase of the cell cycle. Following injury, some CSKs undergo apoptosis, whereas the surviving cells are activated to become stromal fibroblasts (SFs) and myofibroblasts (MyoFBs), as a natural mechanism of wound healing. The SFs and MyoFBs secrete abnormal extracellular matrix proteins, leading to corneal fibrosis and scar formation (corneal opacification). The issue is compounded by the fact that CSK transformation into SFs or MyoFBs is irreversible in vivo, which leads to chronic opacification. In this scenario, corneal transplantation is the only recourse. The application of cell therapy by replenishing CSKs, propagated in vitro, in the injured corneas has been demonstrated to be efficacious in resolving early-onset corneal opacification. However, expanding CSKs is challenging and has been the limiting factor for the application in corneal tissue engineering and cell therapy. The supplementation of serum in the culture medium promotes cell division but inevitably converts the CSKs into SFs. Similar to the in vivo conditions, the transformation is irreversible, even when the SF culture is switched to a serum-free medium. In the current article, we present a detailed protocol on the isolation and propagation of bona fide human CSKs and the morphological and genotypic differences from SFs.

## 1. Introduction

Corneal opacification is predominantly caused by scarring and haze development in the corneal stroma. The haze reduces and distorts the passage of light rays, leading to impaired vision [1,2]. Various factors including but not limited to traumatic injuries, infection, genetic, metabolic and developmental, and idiopathic causes can result in corneal opacification [3,4,5]. Affected individuals require corneal transplantation to restore vision [5]. However, the treatment approach is hindered by the global shortage of donor materials, long-term graft survival, immune response and need for surgical expertise, and high cost [6]. Our pre-clinical study has shown that stromal cell therapy via an intrastromal injection of human corneal stromal keratocytes (CSKs) enabled the repopulation of functional stromal cells in the cornea and reduction in stromal haze/opacities [7]. The possibility of cell propagation in vitro, therefore, can increase the amount of CSKs to achieve a “one donor to multiple recipients” strategy, and in turn, alleviate the issue of donor shortage. In addition, the simple cell delivery via intrastromal injection will be less demanding on surgical expertise and also allow faster visual rehabilitation.

Propagation of CSKs, however, is challenging because CSKs are typically quiescent in adult corneas [8]. To obtain a large number of CSKs for tissue engineering or cell therapy, the primary cells, after isolation from corneal stromal tissue, are often cultured in the presence of fetal bovine serum (FBS), typically in 5–10% concentration depending on the preferred growth stimulation [9,10,11,12,13]. However, this causes CSKs to become fibroblastic, similar to the in vivo corneal wound healing conditions, in that the surviving CSKs are activated to become stromal fibroblasts (SFs) due to the presence of serum proteins and growth factors that are released after the corneal epithelium and basement membrane are structurally compromised. The SFs lose CSK phenotype, which includes its quiescence, dendritic morphology, and expression of keratocan, lumican, and ALDH3A1, and also become proliferative and migratory that are accompanied by the upregulation of stress proteins and fibrosis-related and repair-type extracellular matrix (ECM) proteins (e.g., fibronectin, tenascin-C, and collagen type III), all of which are detrimental to corneal transparency when applied in vivo [10,11]. Although reversal of non-human SFs into CSKs in serum-depleted conditions in vitro resulted in partial recovery of CSK phenotypes, similar attempts to reverse human SFs into CSKs have largely been futile [14,15,16,17]. In many reversal instances, the CSK-specific dendritic morphology with extended cell processes was often lost, and the cells assumed a slender and fibroblastic appearance.

Studies have identified the potential of adult corneal stromal stem cells (CSSCs) in the peripheral cornea and limbus to adopt CSK phenotypes [18,19,20]. The stromal cells harvested near the limbal region, however, are typically heterogeneous. The lack of unique cellular markers makes the isolation of a homogenous and well-defined stem cell population that can differentiate into bona fide CSKs challenging [21,22]. We prefer expanding CSKs from the central corneal stroma. The stromal cell population in the central region is less heterogeneous and predominantly quiescent CSKs. The cells are less prone to fibroblastic transformation at low serum concentrations and easier to maintain while possessing similar wound healing and ECM remodeling properties to the CSSCs. 

Our group has established a culture protocol to propagate human CSKs with a negligible transition to fibroblastic cells [11]. Our method starts with collagenase I digestion of corneal stromal tissue, followed by the culture of primary stromal cells in CSK basal medium with ERI supplement, which contains soluble human amniotic stromal Extract (AME) [23], Rho-associated coiled-coil-forming protein serine/threonine kinase inhibitor (Y-27632) [24], and Insulin-like growth factor 1 [25]. We referred the medium to as CSK complete medium. In the presence of low serum content (0.5% FBS) in the CSK complete medium, the “activated CSKs” can be propagated for 50-80 doublings. These cells have a unique genotype that features an expression of cell proliferation marker (PCNA), reduced expression of CSK-specific proteins (keratocan and lumican), and no expression of fibroblast-associated proteins (fibronectin and tenascin-C) [7,11,26]. They also feature suppressed fibroblast-induced collagen contractibility [11]. Once propagated, the “activated CSKs” culture is placed in serum-free CSK complete medium to drive the cells toward regaining quiescence, dendritic morphology, and unique CSK phenotypes. In the current protocol article, we have shown a step-by-step protocol to isolate, propagate, maintain, and cryopreserve the human CSKs for potential use in corneal tissue engineering and cell therapy.

## 2. Materials and Equipment

### 2.1. Equipment Required

(1) Tissue culture hood;
(2) Germinator (Innotech Bioscience IS-350 Sterilizer; Inotech Bioscience, Rockville, MD, USA);
(3) Rotator (BOECO Rotator Multi Bio RS-24; Boeckel + Co, Hamburg, Germany);
(4) Mortar and pestle;
(5) Mayo scissors (Electron Microscopy Sciences, Hatfield, PA, USA, cat. no. 72996-11) *;
(6) Forceps (Electron Microscopy Sciences, cat. no. 78266-04) *;
(7) Spatula (VWR Scientific, Radnor, PA, USA, cat. no. 82027-528) *;
  (* Note: Autoclave mortar and pestle, scissors, forceps, and spatula before the experiment.);
(8) Surgical blade no. 10 (FEATHER, Osaka, Japan, cat. no. 504169);
(9) Dewar flask;
(10) Weighing balance;
(11) Centrifuge;
(12) Scalpel handle #3 (Electron Microscopy Sciences, cat. no. 72040-03);
(13) Dissecting microscope (Carl Zeiss Stemi 500; Carl Zeiss, Oberkochen, Germany);
(14) Humidified CO2 incubator;
(15) Liquid nitrogen tank;
(16) 40 μm cell strainer (Corning, Corning, NY, USA, cat. no. 352340);
(17) 70 μm cell strainer (Corning, cat. no. 352350);
(18) 15 mL centrifuge tube (Greiner, Kremsmünster, Austria, cat. no. 188271);
(19) 50 mL centrifuge tube (Greiner, cat. no. 210261);
(20) 0.6 mL microcentrifuge tube (Corning, cat. no. MCT-060-C);
(21) 1.5 mL microcentrifuge tube (Corning, cat. no. MCT-150-C);
(22) 60 mm cell culture dish (Corning, cat. no. 353002);
(23) Styrofoam box;
(24) 10 mL syringe (Becton Dickinson, Franklin Lakes, NJ, USA, cat. no. 302149);
(25) Minisart® RC syringe filters (Sartorius, Goettingen, Germany, cat. no. 17764-ACK);
(26) BioCoat™ Collagen I 24-well Clear Flat Bottom TC-treated (Corning, cat. no. 354408);
  Optional: BioCoat™ Collagen I 6-well Clear Flat Bottom TC-treated (Corning, cat. no. 354400);
(27) Mr. Frosty^TM^ freezing container (contains isopropanol) (Thermo Fisher, Waltham, MA, USA, cat. no. 5100-0001);
(28) 2 mL cryovial (Simport, Quebec, Canada, cat. no. T301-2).

### 2.2. Reagents Required

(1) Human amniotic membrane;
(2) Liquid nitrogen;
(3) Ice;
(4) Cadaveric human donor tissue;
(5) Autoclaved 1× phosphate-buffered saline (PBS);
(6) Bovine serum albumin (BSA; Sigma-Aldrich, St. Louis, MO, USA, cat. no. A9418);
(7) Collagenase I (Worthington Biochemical Corporation, Lakewood, NJ, USA, cat. no. CLS-1);
(8) DMEM/F-12 (Invitrogen, Waltham, MA, USA, cat. no. 11330-032);
(9) Deionized water;
(10) MEM insulin-transferrin-selenium (Gibco, Waltham, MA, USA, cat. no. 41400045);
(11) MEM vitamin solution (Gibco, cat. no. 11120052);
(12) MEM amino acids solution (Gibco, cat. no. 11130051);
(13) MEM non-essential amino acids solution (Gibco, cat. no. 11140050);
(14) Antibiotic-antimycotic (Gibco, cat. no. 15240112);
(15) Sodium hydroxide (NaOH; Sigma-Aldrich, cat. no. S2770);
(16) StemMACS^TM^ Y-27632 (Miltenyi Biotec, Bergisch Gladbach, Germany, cat. no. 130-104-169);
(17) Recombinant human insulin-like growth factor 1 (IGF-1; Gibco, cat. no. PHG0078);
(18) L-Ascorbic acid 2-phosphate sesquimagnesium salt hydrate (Sigma-Aldrich, cat. no. A8960);
(19) Fetal bovine serum (FBS; Gibco, cat. no. 10082-147);
(20) StemPro™ Accutase™ cell dissociation reagent (Gibco, cat. no. A1110501); Optional: TripLE Express (Gibco, cat. no. 12604013);
(21) Dimethyl sulfoxide (DMSO; Sigma-Aldrich, cat. no. D2650);
(22) Protein DC Assay (Bio-Rad, Hercules, CA, USA, cat. no. 23235).

## 3. Preparation of Stock and Working Solutions before Procedures

### 3.1. Digestion Buffer Stock and Working Solutions

(1) Weigh 100 mg of collagenase I powder in a 15 mL centrifuge tube.
(2) Add 8 mL of 1× PBS and agitate the tube to dissolve the powder.
(3) Fill up the remaining 2 mL to make a final volume of 10 mL of 10 mg/mL collagenase type I.
(4) Filter sterilize the collagenase I solution using Minisart^®^ RC syringe filter that is attached to a 10 mL syringe. Aliquot sterile collagenase I at 1 mL each into 1.5 mL microcentrifuge tubes and store the tubes of stock solutions at −20 °C * (* Note: Once thawed, collagenase should be stored at 4 °C. Repeat freeze–thawing should be avoided.)
(5) Prepare 10 mL of digestion buffer working solution, containing 1 mg/mL collagenase I and 0.1% BSA by adding 1 mL of 10 mg/mL collagenase (prepared in Steps 1–5 above) and 10 mg of BSA to 9 mL of CSK basal medium which preparation protocol will be described in the following section *, **.
  (* Note: The digestion buffer working solution should be prepared fresh before cadaveric donor corneal tissue dissection.)
  (** Note: Adjust the volume of the digestion buffer accordingly based on the amount required for the corneal dissection procedure on the day. A central stromal tissue from one human cornea can typically be digested in 1 mL of digestion buffer working solution.)

### 3.2. CSK Basal Medium

(1) Add 47.45 mL of DMEM/F-12 to a 50 mL centrifuge tube.
(2) Add 50 μL of 100× MEM insulin-transferrin-selenium to the tube *.
(3) Add 500 μL of 100× MEM non-essential amino acids solution to the mixture *.
(4) Add 500 μL of 100× MEM vitamin solution to the mixture *.
(5) Add 500 of 100× antibiotic-antimycotic to the mixture *.
(6) Add 1 mL of 50× MEM amino acids solution to the tube and gently mix it *.
  (* Note: Step 2 dilutes the reagent to 0.1x concentration. Steps 3–6 dilute the reagents to 1x concentration.)
(7) Adjust pH with 1N NaOH dropwise to ~pH7 (the media will change from yellow to orange-red, similar to the stock DMEM/F-12).

### 3.3. 100 μg/mL IGF-1

(1) Add 200 μL of 1× PBS to 20 μg of IGF-1 in a 1.5 mL microcentrifuge tube.
(2) Resuspend by pipetting the solution up and down until the powder is dissolved.
(3) Store at 4 °C until use.

### 3.4. 10 mM ROCK Inhibitor (Y-27632)

(1) Dissolve 2 mg of StemMACS^TM^ Y-27632 in 624.4 μL of DMSO in a 1.5 mL microcentrifuge tube.
(2) Resuspend by pipetting the solution up and down until the powder is dissolved.
(3) Aliquot 100 μL of 10 mM StemMACS^TM^ Y-27632 into 0.6 mL microcentrifuge tubes.
(4) Store at −20 °C until use *.
  (* Note: Once thawed, store the solution at 4 °C and avoid repeat freeze–thawing.)

### 3.5. 50 mM L-ascorbic 2-phosphate

(1) Weigh 145 mg of L-ascorbic-2-phosphate sesquimagnesium salt hydrate powder into a 15 mL centrifuge tube.
(2) Add 8 mL of deionized water and mix until the powder is dissolved.
(3) Fill the tube to the 10 mL mark with deionized water.
(4) Filter the L-ascorbic 2-phosphate solution using a Minisart^®^ RC syringe filter attached to a 10 mL syringe.
(5) Aliquot 1 mL of the filtered solution to 1.5 mL microcentrifuge tubes.
(6) Store at −20 °C until use *.
  (*Note: Once thawed, store the solution at 4 °C and avoid repeat freeze–thawing.)

### 3.6. CSK Complete Medium

(1) Add 9.6 mL of CSK basal medium in a 15 mL centrifuge tube.
(2) Add 1 μL of 100 μg/mL IGF-1 to the tube to obtain a final concentration of 10 ng/mL *.
(3) Add 10 μL of 10 mM Y-27632 to obtain a final concentration of 10 μM *.
(4) Add 50 of heat-inactivated FBS to obtain a final concentration of 0.5% *.
(5) Add 100 μL of 50 mM L-ascorbic 2-phosphate to obtain a final concentration of 0.5 mM *.
(6) Add 5 μg/mL of AME and mix well (the AME preparation protocol will be elaborated in the Protocol section) *.
  (* Note: All supplements/growth factors should be added immediately before culture).

## 4. Detailed Procedure

### 4.1. Overview of CSK Culture Procedure

**Figure 1:**
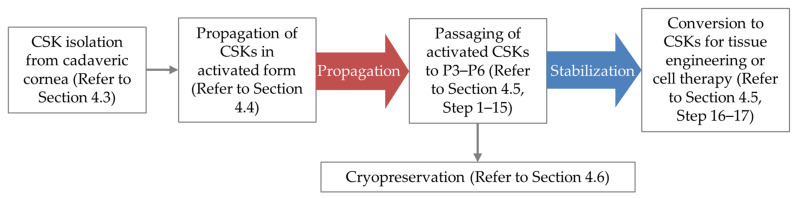
Overview of human corneal stromal keratocyte (CSK) cell culture procedure. In the propagation phase, the culture medium is supplemented with 0.5% fetal bovine serum (FBS) to support the proliferation of CSKs, which are otherwise quiescent. In the stabilization phase, the FBS is removed from the culture medium to allow the cells to regain bona fide CSK phenotypes.

The culture procedure should be initiated with the preparation of amnion extract, which takes 2 days at the most. Figure 1 displays the overview of the CSK isolation from donor corneal tissue to the conversion of activated CSKs into CSKs for cell-based therapies. Following amniotic protein extraction, the isolation of CSKs from cadaveric corneal tissue may proceed, which takes 1 day to complete. In the propagation phase, the isolated CSKs are cultured in CSK complete medium (containing 0.5% FBS) up to P6. Cryopreservation of activated CSKs can be carried out at any time between P3 and P6 (inclusive) when the cells reached ~70% confluency. For cell therapy applications, the activated CSKs at P3–P6 are converted to CSKs by subjecting the cells to the stabilization phase in CSK complete medium (without 0.5% FBS) for 7–21 days.

### 4.2. Extraction of Proteins from Amniotic Membrane

The human amniotic membrane in the form of extract has been widely used to facilitate the propagation of various cell types, including corneal epithelial cells and CSKs [23,27,28,29,30]. The effects of AME in the CSK culture system are multifactorial. The extract has been shown to be paramount in preventing fibroblastic transformation via the suppression of the TGF-β1/β2 pathway and is critical to eliminate fibroblast growth and maintain CSK characteristics in vitro [11,30,31,32]. The amniotic membrane is also known to exert immunomodulatory effects, secrete proteins that support ECM and cytoskeleton remodelling, and modulate cell–matrix interactions that may be crucial in maintaining CSK phenotypes [11,33,34,35,36]. 

AME can be derived either from fresh or cryopreserved human amniotic membrane; however, it is more likely for any lab to obtain a cryopreserved amniotic membrane. Amniotic membrane is typically cryopreserved in medium containing DMEM with 50% glycerol and penicillin/streptomycin [37]. We have shown that CSK cultures supplemented with extracts from either fresh or cryopreserved amnion samples resulted in similar cell morphology and viability, with only a marginal reduction in cell proliferation when AME from cryopreserved tissue was used [23]. We acknowledge that procurement of human amniotic membrane can be challenging in some countries. Sterilized γ-irradiated amniotic membrane is commercially available but its effect on the CSK culture has not been explored [38].

(Note: All procedures that require manipulation of amniotic membrane or its extract should be carried out in a tissue culture hood, with exceptions; the rotation and centrifugation steps. Since AME for cell culture purpose has to be prepared without the presence of protease inhibitors, all extraction steps must be kept in cool temperature or on ice to prevent intracellular enzyme activation, protein denaturation, and degradation.)

(1) For fresh amniotic membrane, proceed to step 5.
(2) For cryopreserved amniotic membrane, thaw the vial containing the amniotic membrane at 4 °C overnight.
(3) Prepare the tissue culture hood by UV sterilization for 15 min, followed by wiping off the work surface with 70% ethanol.
(4) Turn on the germinator and heat-sterilize the forceps, scissors, and spatula. Ensure that forceps, scissors, and spatula have cooled down before handling them.
(5) Wash the amnion 3–5 times in 1× PBS to remove traces of blood (for fresh amnion) or glycerol (for cryopreserved amnion). More washes have to be carried out if the amnion still has traces of blood.
(6) Drain away PBS by squeezing the amnion with the forceps in a downward motion. Repeat this a few times to remove as much PBS as possible for an easier grinding process later (PBS retention can cause ice formation in the amnion tissue when cooled with liquid nitrogen, leading to difficulty in grinding).
(7) Place the amnion in a 60 mm cell culture dish and using mayo scissors, cut it into ~1 cm^2^ pieces.
(8) Add liquid nitrogen to the mortar and pestle to cool it down.
(9) Transfer 5–8 amnion pieces to the cooled mortar and add liquid nitrogen.
(10) Grind the cooled amnion pieces using pestle into “powder” *, **, ***.
  (* Note: “Powder” is used as a term. The amnion will not be fully powderized. The grinding process only shears it into smaller fragments).
  (** Note: Due to the water retained by the membrane, the tissue will be hardened upon the addition of liquid nitrogen and challenging to grind. Allow the liquid nitrogen to evaporate before grinding to soften the amnion somewhat.)
  (*** Note: Before the membrane starts to become too soft, refill the mortar with liquid nitrogen. Do not let the membrane thaw fully.)
(11) Transfer the amnion “powder” to a pre-weighed 50 mL centrifuge tube. Repeat Steps 8–11 until all amnion pieces have been processed.
(12) Weigh the 50 mL centrifuge tube containing the amnion “powder” and mark down the weight of the “powder”.
(13) Add 3 mL of 1× PBS into the tube per gram of amnion “powder”.
(14) Place the tube on a rotator at a speed of 100 rpm at 4 °C for 48 h.
(15) After 48 h, filter the suspension through a 70 μm cell strainer.
(16) Collect the flow-through filtrate and centrifuge at 3000 rpm for 15 min at 4 °C.
(17) Aliquot the supernatant into 1.5 mL microcentrifuge tubes and centrifuge the tubes at 12,000× *g* for 15 min at 4 °C.
(18) Collect the clear supernatant (containing AME) and aliquot the supernatant 1 mL each into new 1.5 mL microcentrifuge tubes *.
  (* Note: Remove 200 μL of the AME filtrate from one of the microcentrifuge tubes for protein assay. Protein concentration can be measured with a Bio-Rad Protein DC Assay according to the manufacturer’s protocol).
(19) Store the AME aliquots at −80 °C until use.

### 4.3. Isolation of CSKs from Cadaveric Donor Cornea

**Figure 2:**
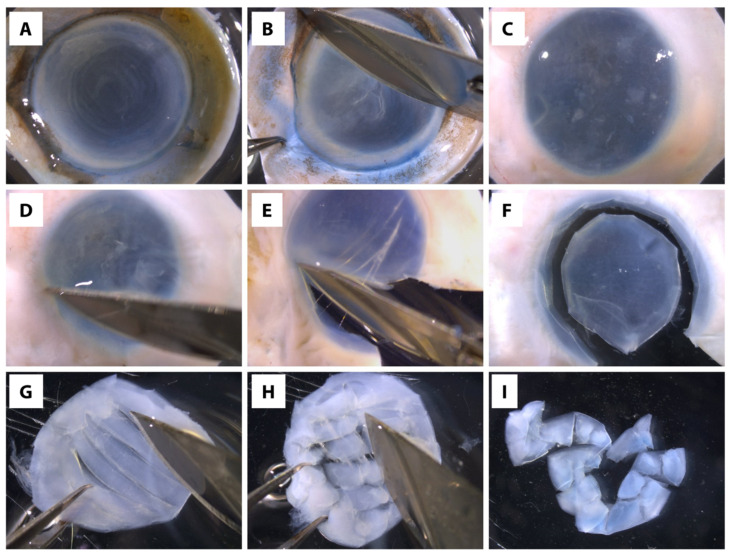
Still photographs of the human corneal tissue dissection. (**A**–**D**) The first dissection steps involve the removal of the corneal epithelial and endothelial cells, and trabecular meshwork from the corneas, by scraping with a surgical blade no. 10. (**E**,**F**) The corneal stroma is then separated from the scleral tissue by cutting ~2 mm from the sclera. (**G**–**I**) Finally, the corneal stroma is cut into smaller pieces by leaving the edges of each piece still attached to the adjacent pieces.

(1) Place the forceps and surgical blade no. 10 with its holder in the tissue culture hood.
(2) UV-sterilize the hood for 15 min and clean the work surface with 70% ethanol.
(3) Heat sterilize the surgical instruments (forceps and blade holder using germinator and cool them down before dissecting the corneal tissues).
(4) Fill 3 mL of 1× PBS each into four 60 mm tissue culture dishes.
(5) Place the cadaveric donor corneal tissue in one of the 60mm culture dishes.
(6) Scrape off the corneal epithelial and endothelial cells, as well as trabecular meshwork (if any) from the donor tissue using the surgical blade (Figure 2A–D) *, **, ***.
  (* Note: Important to ascertain the complete removal of corneal epithelial and endothelial cells because these cells grow faster than CSK in culture, and consequently, generating culture with mixed cell types.)
  (** Note: If difficulties are encountered in removing the corneal epithelial cells, the epithelial scraping can be aided by dipping the cornea in 20 mg/mL dispase solution in DMEM/F-12 for 30 min at 37 °C
  (*** Note: If isolating CSKs from an eye globe, use mayo scissors to cut around the globe to remove the majority of the sclera, leaving ~3 mm of sclera around the cornea. Scrape top and bottom of the tissue to remove unwanted tissue/cells, such as the epithelial cells, endothelial cells, trabecular meshwork, and iris/ciliary tissues before proceeding to Step 7.)
(7) Place the tissue in a new 60 mm culture dish containing 1× PBS.
(8) Heat sterilize the surgical instruments and cool them down.
(9) Isolate the central clear stroma (7–8 mm diameter) without including any limbal or scleral tissue (Figure 2E,F).*
  (* Note: Important to ascertain the complete removal of scleral tissue because the scleral fibroblasts can contaminate the CSK culture.)
(10) Place the isolated stroma in a new 60 mm culture dish containing 1× PBS and continue to scrap both surfaces to ensure complete removal of epithelium and endothelium, and no tissues other than the stroma.
(11) Heat sterilize the surgical instruments and cool them down.
(12) Place the stromal tissue in a new 60 mm culture dish containing 1× PBS and cut the tissue into ~1 mm 2 pieces with a surgical blade no. 10 (Figure 2G–I) *.
  (* Note: It is recommended to leave the edges intact as this allows easier transfer of tissue to the digestion buffer and also to prevent excessive tissue tearing to preserve the CSK viability in the tissue.)
(13) Transfer the cornea to a 15 mL centrifuge tube containing 1 mL of digestion buffer working solution.
(14) Incubate the tube at 37 °C in a 5% CO_2_ humidified incubator for 10–12 h or until ~90% of tissue has been digested. Do not exceed 16 h because it can be detrimental to the cell viability.

### 4.4. Culture of Human CSKs

**Figure 3:**
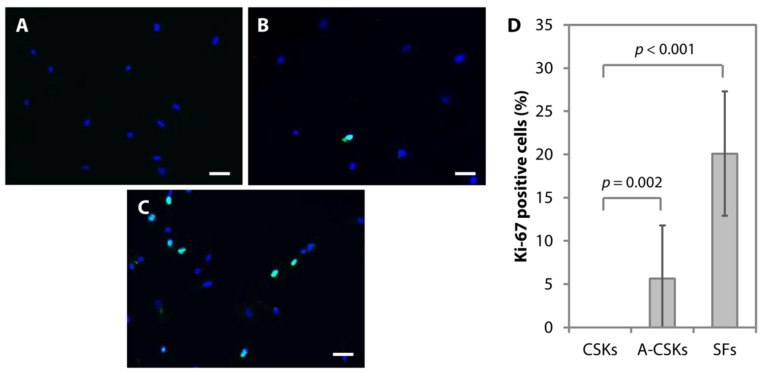
Proliferative capacity of corneal stromal keratocytes (CSKs), activated CSKs (A-CSKs), and stromal fibroblasts (SFs). The representative images of CSKs (**A**), A-CSKs (**B**), and SFs (**C**) were captured from cells at P5, which were expanded from the same donor. (**D**) The proliferative capacity (indicated by Ki-67-positive cells/total number of cells × 100%) of the A-CSKs was 5.6 ± 6.1%. On day 14, following medium switching to serum-free conditions, the proliferative capacity of CSKs was 0%. The SFs had a significantly higher proliferation rate of 20.1 ± 7.2% compared to both the CSKs (*p* = 3.55 × 10^−8^) and the activated CSKs (*p* = 3.78 × 10^−5^). Group comparisons were statistically determined using one-way ANOVA and Tukey comparison tests. Scale bars = 100 μm.

Similar to in vivo, the CSKs isolated from human donor corneas inherently retain their quiescence nature in vitro. Hence, to produce sufficient number of CSKs for clinical translational purposes, 0.5% FBS has to be added to the culture medium (CSK complete media) to allow cell propagation (Figure 3B,D). The propagation capacity of the cells, referred to as the activated CSKs, can be captured by the immunofluorescence staining of Ki-67. Before applying the cells for tissue engineering or cell therapy (i.e., intrastromal CSK injection), the activated CSKs have to be returned to quiescence (Figure 3A,D). Hence, the cells are cultured in the CSK complete medium without FBS for 7–21 days to allow the cells to regain bona fide CSK morphology and phenotypes. We refer these cells to as the CSKs. Adding 5% FBS to the culture medium, significantly increase the cell proliferation but irreversibly transform the cells into SFs (Figure 3C,D).

(1) Filter the tissue digest from the preceding section (Isolation of CSKs from cadaveric donor cornea) using a 40 μm cell strainer into a 50 mL centrifuge tube to remove undissociated materials. Following that, wash the tube with 10 mL of 1× PBS once and pass it through the filter.
(2) Add 1× PBS to at least 3 times the volume of the filtrate to dilute the collagenase I.
(3) Centrifuge the solution at 350× *g* for 7 min at room temperature.
(4) Remove the supernatant *.
  (* Note: Do not disturb the cell pellet. Because the number of CSKs is typically low, the cell pellet may not be visible.)
(5) Resuspend the cell pellet and add 3 mL of 1× PBS.
(6) Centrifuge the solution at 350× g for 7 min at room temperature.
(7) Remove the supernatant *.
  (* Note: It is important to ascertain that the collagenase I solution is completely removed before culture. If necessary, repeat Steps 5–7 for complete removal of collagenase I from the system.)
(8) Add CSK complete medium to the tube and disperse the cell pellet by gentle tapping of the tube.*
  (* Note: The volume of medium to be added depends on the number of corneas that are processed. Cells isolated from one cornea are typically cultured in one well of a BioCoat™ Collagen I 24-well plate. As a guide, 1 mL of medium is usually sufficient for a well of the culture plate.)
(9) Seed the cells in the BioCoat™ Collagen I 24-well plate.
(10) Incubate the cells at 37 °C in a 5% CO_2_ humidified incubator.
(11) Check the activated CSKs under the microscope and change the CSK complete medium every 3–4 days *, **, ***, ****.
  (* Note: Cells may take up to 1 week to adhere and start extending their cellular processes. If cells do not grow, continue to observe for up to 3 weeks.)
  (** Note: For P0 cells, it may take between 2 weeks and 1 month to reach ~70% confluency.)
  (*** Note: During the first medium change, aspirate the medium with floating cells and transfer it into a new well. These floating cells may still be viable and would attach to the surface of the new well. Add CSK complete medium into the well and observe the cells every 3–4 days.)
  (**** Note: If the cell number is low, especially during the first week of culture, perform half medium change, i.e., remove 50% volume of the medium from the well and replenish with fresh CSK complete medium of the same volume.)

### 4.5. Passaging of Human CSKs and Preparation for Cell Therapy

The activated CSKs should be passaged once ~70% confluency is reached. The morphology of cells tends to change into fibroblastic once the confluency exceeds 80%. This section of the protocol is also used to harvest CSKs in the preparation for tissue engineering or cell therapy applications.

(1) Aspirate the medium from the culture well.
(2) Wash the well with 1× PBS twice.
(3) Add StemPro™ Accutase™ cell dissociation reagent to the well *.
  (* Note: Ensure that cell dissociation reagent fully cover the well.)
(4) Incubate for 3 min at room temperature *, **.
  (* Note: Do not leave the cells in the StemPro™ Accutase™ cell dissociation reagent for a prolonged period. If longer incubation is needed, check under microscope every 1 min and stop the reaction once ~90% of cells are detached.)
  (** Note: If using TrypLE Express, incubate the cells at 37 °C in a 5% CO_2_ humidified incubator for 3 min.)
(5) Add 1× PBS to at least 3 times the volume of the StemPro™ Accutase™ cell dissociation reagent into the well and flush the well by pipetting up and down to ensure most cells are detached and collected.
(6) Pipette the solution containing activated CSKs or CSKs into a 15 mL centrifuge tube.
(7) Centrifuge the tube at 350× g for 7 min at room temperature.
(8) Remove the supernatant.
(9) Resuspend the cell pellet in 3 mL of 1 × PBS.
(10) Centrifuge the tube at 350× g for 7 min at room temperature.
(11) Remove the supernatant.
(12) For passaging, resuspend the activated CSK pellet in CSK complete medium. Tap the tube lightly to disperse the pellet into a single-cell suspension *.
  (* Note: For sub-culturing in BioCoat™ Collagen I 24-well plate, add 1 mL of medium per well. For a 6-well plate, add 1.5ml–2ml of medium per well. Proceed to Step 13 to continue the passaging procedure. Proceed to Step 16 to convert activated CSKs into CSKs for tissue engineering or cell therapy applications.)
(13) Pipette the solution containing cells into the well.
(14) Incubate the plate at 37 °C in a 5% CO_2_ humidified incubator.
(15) Change the media every 2–3 days.
(16) To convert into CSKs, activated CSKs at ~70% confluency at P3–P6 are maintained in the CSK complete medium (without 0.5% FBS) for 7–21 days. Observe the morphology of the cells every 3–4 days. Cells that do not assume long cellular processes and distinct cell bodies are disqualified from further applications. When the required cell number and desired cell morphology are met, single-cell suspension of CSKs can be prepared by repeating Step 1–11 above.
(17) Resuspend the CSK in 1x PBS. Tap the tube lightly to disperse the pellet into a single-cell suspension *.
  (* Note: To prepare CSKs for cell therapy in vivo, determine the cell number with a hematocytometer or an automated cell counter and calculate the required therapeutic density of the cells before adding the 1× PBS in the tube. Apply the cells in vivo within 30 min of preparation.)

### 4.6. Cryopreservation of Human CSKs

Cryopreservation of the activated CSKs should be performed when at least 4 wells of a 6-well plate have reached a confluency of ~70% and when they are not planned for any immediate experiments. This is to minimize the passage number and thus, the propensity of cells to differentiate into fibroblasts at higher passage numbers.

(Note: Approximately 30–50% of frozen primary cells may lose viability after thawing. Therefore, it is not recommended to freeze down cells when the cell number is too low, such as less than 4-well of 6-well plate or less than 250,000 cells.)

(1) Aspirate the culture medium from the well.
(2) Wash the well with 1× PBS twice.
(3) Add 200 μL (for 24-well plate) or 400 μL of StemPro™ Accutase™ cell dissociation reagent (for 6-well plate) into each well.
(4) Incubate for 3 min at room temperature *, **.
  (* Note: Do not leave the cells in the StemPro™ Accutase™ cell dissociation reagent for a prolonged period. If longer incubation is needed, check under microscope every 1 min and stop the dissociation reaction once ~90% of cells have been detached.)
  (** Note: If using TrypLE Express, incubate the cells at 37 °C in a 5% CO_2_ humidified incubator for 3 min.)
(5) Add 1× PBS at least 3 times the volume of StemPro™ Accutase™ cell dissociation reagent into the well and flush the well by pipetting up and down to detach the cells.
(6) Collect the cell suspension into a 15 mL centrifuge tube.
(7) Centrifuge the tube at 350× *g* for 7 min at room temperature.
(8) Discard the supernatant.
(9) Resuspend the cell pellet in 3 mL of 1× PBS.
(10) Centrifuge the tube at 350× *g* for 7 min at room temperature.
(11) Discard the supernatant.
(12) Resuspend the cell pellet with 450 μL of CSK complete medium.
(13) Add 50 μL of DMSO to obtain a final 10% DMSO concentration in the cell suspension.
(14) Transfer the cell suspension into a 2 mL cryovial.
(15) Place the cryovial in a Mr. Frosty^TM^ freezing container and freeze it at −80 °C for at least 24 h.
(16) Transfer the cryovial under liquid nitrogen for long-term storage.

## 5. Expected Results

### 5.1. Cell Morphology

**Figure 4:**
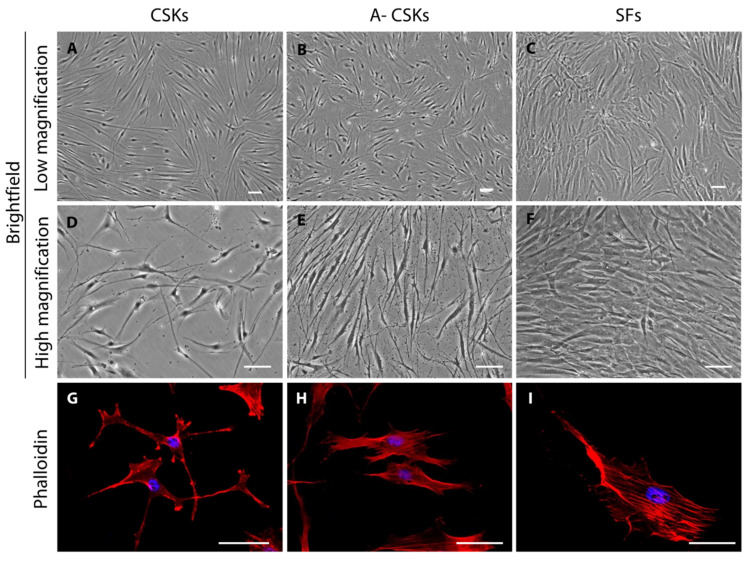
Morphology of corneal stromal keratocytes (CSKs), activated CSKs (A-CSKs), and stromal fibroblasts (SFs). The representative images were captured from cells at P5, which were expanded from the same donor. (**A**–**F**) Brightfield images at low and high magnification revealed the loss of thin, dendritic morphology and long cellular processes, typically seen in the CSKs, in the SFs. The SFs also featured larger cell bodies compared to the CSKs and A-CSKs. (**G**–**I**) Phalloidin staining showed the stellate morphology of the CSKs, which was progressively lost in the A-CSK and SF cell culture. Scale bars = 100 μm.

The differences in the cell morphology can be identified using phase-contrast microscopy. The human CSKs show thinner and distinctly more round-ish cell bodies (Figure 4A,D) than the activated CSKs (Figure 4B,E). The CSKs also feature longer cellular processes than the activated CSKs. The SFs, which are converted from the CSKs by the addition of 5% FBS in the CSK complete medium, show broader cell bodies with shorter cellular processes than both the CSKs and the activated CSKs (Figure 4C,F). Further confirmation of phenotypic reversal of the cells can be achieved by staining with phalloidin. The human CSKs (Figure 4G) assume a more stellate morphology with a concentrated F-actin organization near the end of the cellular processes compared to the activated CSKs (Figure 4H). In stark contrast, the SFs are slender in shape and have a more bipolar morphology with larger cell bodies and an absence of cellular processes (Figure 4I).

### 5.2. Protein and Gene Expression

**Figure 5:**
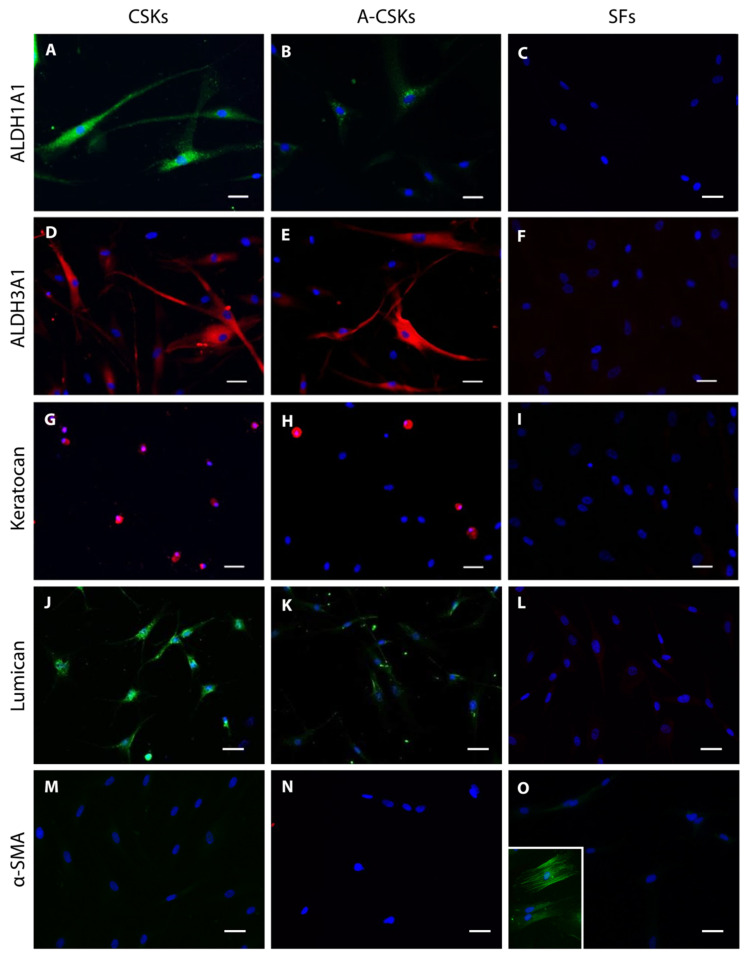
Protein expression of corneal stromal keratocytes (CSKs), activated CSKs (A-CSKs), and corneal fibroblasts (SFs). (**A**,**D**,**G**,**J**) Typical CSK markers, such as ALDH1A1, ALDH3A1, keratocan, and lumican were strongly expressed in the CSKs following 14 days of culture media switching to serum-free conditions. (**B**,**E**,**H**,**K**) In the propagation medium, the A-CSKs exhibited an attenuated expression of the CSK markers. (**C**,**F**,**I**,**L**) In contrast, in medium supplemented with 5% FBS, the SFs did not express or express only a little of the CSK markers. (**M**,**N**,**O**) All three cell types were not immunoreactive with α-smooth muscle actin (α-SMA), the cell marker of corneal stromal myofibroblasts (see inset in pane O). Scale bars = 50 μm.

The specific marker expression can be validated with immunofluorescence staining and real-time polymerase chain reaction (RT-PCR), which includes ALDH1A1, ALDH3A1, keratocan, and lumican (the antibodies, primers, and test procedures can be found in our previously published articles) [11,26]. The CSKs strongly express ALDH1A1 (Figure 5A), ALDH3A1 (Figure 5D), keratocan (Figure 5G), and lumican (Figure 5J), while the activated CSKs typically have an attenuated expression of these markers (Figure 5B,E,H,K). This is attributed to the activated CSKs being cultured in the media containing 0.5% FBS for a prolonged period. When the cells from the same donor are cultured in CSK complete medium containing 5% FBS, the cells turn fibroblastic and lose the immunoreactivity to ALDH1A1 (Figure 5C), ALDH3A1 (Figure 5F), keratocan (Figure 5I), and lumican (Figure 5L). The CSKs (Figure 5M), A-CSKs (Figure 5N), and SFs (Figure 5O) do not stain α-smooth muscle actin (α-SMA), the cell marker of MyoFBs (Figure 5O inset shows typical α-SMA staining in the stress fibers of MyoFBs). The MyoFBs are converted from SFs by the addition of TGF-β1.

**Figure 6:**
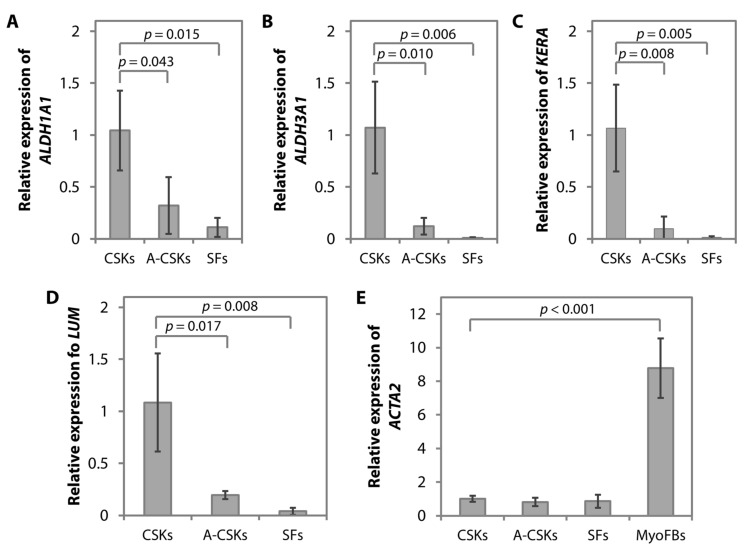
Gene expression of corneal stromal keratocytes (CSKs), activated CSKs (A-CSKs), and corneal fibroblasts (SFs). The gene expression was detected using real time-polymerase chain reaction. Similar to the protein expression, CSK-associated genes, such as *ALDH1A1* (**A**), *ALDH3A1* (**B**), *KERA* (**C**), and *LUM* (**D**) were strongly expressed in the CSKs following 14 days of culture media switching to serum-free conditions and were significantly upregulated when compared to the A-CSKs and SFs. (**E**) The corneal stromal myofibroblast (MyoFB)-associated gene, *ACTA2*, was significantly downregulated in the CSKs, A-CSKs, and SFs compared to the MyoFBs. For the analysis of differentially expressed genes, CSKs was used as the reference group for comparison, whereas GAPDH was used as the housekeeping gene. Group comparisons were statistically determined using one-way ANOVA and Tukey comparison tests.

The differences in the ALDH1A1, ALDH3A1, keratocan, and lumican protein expression in the three cell types are usually reflected similarly in their gene expression. On RT-PCR, the expression of *ALDH1A1* (Figure 6A), *ALDH3A1* (Figure 6B), *KERA* (Figure 6C), and *LUM* (Figure 6D) is significantly lower in the SFs than in the CSKs. Similar to the protein expression found in the immunofluorescence staining, the activated CSKs display an intermediate expression of *ALDH1A1* (Figure 6A), *ALDH3A1* (Figure 6B), *KERA* (Figure 6C), and *LUM* (Figure 6D). No significant difference in the level of *ACTA2* expression is found in the CSKs, A-CSKs, and SFs (Figure 6E). *ACTA2* in all three cell types is significantly downregulated compared to MyoFBs (*p* = 3.16 × 10^−5^ vs. CSKs; *p* = 2.63 × 10^−5^ vs. A-CSKs; and *p* = 2.74 × 10^−5^ vs. SFs) (Figure 6E).

### 5.3. Troubleshooting Guide

During the isolation of CSKs from donor corneal stroma, reagent preparations, and the culture of CSKs, some problems described in Table 1 may be encountered. The possible explanations and solutions are included in Table 1 to aid researchers in troubleshooting the problems.

## 6. Conclusions

Besides describing the step-by-step procedures to isolate, culture, and propagate human CSKs, the current article also highlighted the complexity of CSK culture protocol, compared to the SF culture, which can be propagated in a simple medium that contains only DMEM/F-12 and 5% FBS [12,39,40,41]. In addition, this article also highlighted the morphological and protein and gene expression differences between CSKs and SFs. The stark differences in the cellular morphology and phenotypes emphasized why the two cell types should not be used interchangeably in the literature and should not be therapeutically introduced in vivo before proper cell culture and propagation methods have been followed. Cell therapy with incorrect cell type could be detrimental to the patient’s vision as we have shown in our rat experimental model [7]. With this protocol, we have greatly improved the efficiency of propagating CSKs and we can now increase the number of bona fide CSKs to achieve a “one donor to multiple recipients” strategy, and in turn, alleviate the issue of donor shortage. Our work suggests the plausibility of tissue engineering and cell-based therapy for treating corneal stromal disorders.

## Figures and Tables

**Table 1:** Potential problems that may arise during CSK culture and their respective solutions.

Problem Encountered	Explanations	Solutions
Low amount of total protein in AME	Amnion proteins are degraded	• Ensure that the human amnion is placed in 4 °C or on ice before and during extraction.
		• Store AME immediately after extraction procedure at −80 °C and thaw only when required.
	Loss of proteins during processing	• Ensure that amnion is processed as soon as possible from the time of collection/harvest.
	The sample is diluted with a large volume of PBS after grinding	• After filtering, wash the strainer with sufficient PBS to ensure most soluble proteins are collected.
		• Reduce the volume of PBS added to the tube containing the AME “powder”.
	The sample is obtained from the anterior part of the amnion sac where the stroma is the thinnest	• Collect protein from the more posterior part with thicker stroma; however, avoid collecting the vascularized tissue.
Corneal tissue is not or only partially digested	Collagenase I might have been degraded	• Prepare collagenase I digestion fresh.
		• Ensure proper storage of the stock solution, as well as the collagenase I powder.
	Insufficient digestion	• Prolong the incubation time but do not exceed 24 h in 0.1% collagenase I.
		• A higher concentration of collagenase I (0.2–0.3%) can be used to digest the corneal tissue but do not subject the tissue to digestion exceeding 10 h
Epithelial cell contamination in CSK culture	Insufficient scraping of the anterior (epithelial side) of the cornea	• Check under the microscope to ensure that sufficient scraping of the corneal epithelial cells, including the limbal area, has been performed.
		• If difficulties in removing the epithelial cells from the cornea are encountered, pre-treatment with dispase could be performed.
	Bacterial contamination	• Place the surgical instruments used in the corneal dissection in the germinator for at least 30 s before each step.
	Epithelial cell growth in culture	• Ensure that limbal epithelial cells, if any, are completely removed from the sclera.
		• Cut ~2mm into the cornea, away from the sclera to ensure no contamination from the limbal epithelial cells.
		• Partial trypsinization can be performed in culture. Epithelial cells generally take longer (5–7 min) to be detached from the plate in the presence of dissociation reagent, compared to the CSKs (3–5 min). Earlier termination of the dissociation reactions would allow the majority of the CSKs to be lifted, while the epithelial cells are still attached to the plate.
CSKs are not viable	Status/condition of the donor cornea is not optimal for culture (i.e., donor’s age, disease status, etc.)	• Obtain only healthy corneas from younger donors (set a cutoff age of 70 years old), if possible. The younger the donors, the higher the number of viable cells.
	Prolonged storage of donor cornea in Optisol before CSK isolation	• Process the cornea as soon as possible upon receiving the tissue.
	A prolonged period of tissue digestion in collagenase I	• Do not exceed 24 h of digestion in 0.1% collagenase I.
		• If using higher concentrations of collagenase (0.2–0.3%), do not exceed 10 h of incubation.
	Reagents used in the culture are not in optimal conditions	• Ensure that the correct concentrations of reagents are prepared.
		• Ensure that the reagents have not expired.
		• Ensure reagents are stored correctly and according to the manufacturers’ instructions.
		• Reagents may have degraded. Prepare the stock solutions fresh.
		• Always prepare CSK media (with supplements) fresh. If prepared in advance, keep it at 4 °C for no more than one week.
Microbial contamination	Aseptic techniques are not observed	• Ensure that work surfaces are wiped with 70% ethanol, surgical instruments are heat-sterilized and all materials and reagents used are sterile.
		• If contamination is minimal, remove the media and wash thrice with 1× PBS with antibiotic-antimycotic. Add media containing 2× antibiotic-antimycotic.
	Donor corneal tissue is infected	• If the cornea looks cloudy/hazy, do not process.

## Data Availability

Not applicable.
